# Evolution Analysis of Simple Sequence Repeats in Plant Genome

**DOI:** 10.1371/journal.pone.0144108

**Published:** 2015-12-02

**Authors:** Zhen Qin, Yanping Wang, Qingmei Wang, Aixian Li, Fuyun Hou, Liming Zhang

**Affiliations:** 1 Crop Research Institute, Shandong Academy of Agricultural Sciences, Jinan, China; 2 Shandong Key Laboratory of Animal Disease Control and Breeding/Institute of Animal Science and Veterinary Medicine, Shandong Academy of Agricultural Sciences, Jinan, China; National Cheng-Kung University, TAIWAN

## Abstract

Simple sequence repeats (SSRs) are widespread units on genome sequences, and play many important roles in plants. In order to reveal the evolution of plant genomes, we investigated the evolutionary regularities of SSRs during the evolution of plant species and the plant kingdom by analysis of twelve sequenced plant genome sequences. First, in the twelve studied plant genomes, the main SSRs were those which contain repeats of 1–3 nucleotides combination. Second, in mononucleotide SSRs, the A/T percentage gradually increased along with the evolution of plants (except for *P*. *patens*). With the increase of SSRs repeat number the percentage of A/T in *C*. *reinhardtii* had no significant change, while the percentage of A/T in terrestrial plants species gradually declined. Third, in dinucleotide SSRs, the percentage of AT/TA increased along with the evolution of plant kingdom and the repeat number increased in terrestrial plants species. This trend was more obvious in dicotyledon than monocotyledon. The percentage of CG/GC showed the opposite pattern to the AT/TA. Forth, in trinucleotide SSRs, the percentages of combinations including two or three A/T were in a rising trend along with the evolution of plant kingdom; meanwhile with the increase of SSRs repeat number in plants species, different species chose different combinations as dominant SSRs. SSRs in *C*. *reinhardtii*, *P*. *patens*, *Z*. *mays* and *A*. *thaliana* showed their specific patterns related to evolutionary position or specific changes of genome sequences. The results showed that, SSRs not only had the general pattern in the evolution of plant kingdom, but also were associated with the evolution of the specific genome sequence. The study of the evolutionary regularities of SSRs provided new insights for the analysis of the plant genome evolution.

## Introduction

Plant genomes are filled with low-complexity repetitive sequences. One of the most frequent low complexity sequences is simple sequence repeats (SSRs, defined as1~6 bp unit) [1]. Studies have shown that SSRs have many important biological functions, such as the regulation of chromatin organization, DNA metabolic processes, gene activity and RNA structure [2–4]. SSRs have therefore emerged as the third major class of genetic variations, alongside copy number variations and single nucleotide polymorphisms [5].

SSRs in plant genome sequences evolve along with the plant gene and genome evolution. Gene and genome duplications are major driving forces of gene diversification and evolution [6]. Angiosperms are paleopolyploids, that is to say the genome of their common ancestor was subject to a large-scale or even genome wide duplication event during the Late Jurassic or Early Cretaceous, 100~160 million years ago [7–8]. This duplication event might have triggered the angiosperm radiation during the Late Cretaceous, which is apparent in fossil record [9]. There are evidences for several other large-scale or genome-wide duplication events among the angiosperms [8, 10–17]. The core eudicotyledon apparently duplicated their genomes in the Late Cretaceous, while the common ancestor of the *Brassicales* did so again in the Cenozoic [8, 18]. Moss *P*. *patens* is a paleopolyploid as well. The genome duplication to have occurred between 30 and 60 million years ago [19]. Interestingly, the retention of genes after such large-scale duplication events has been shown to be biased towards certain functional classes [20–22]. It has been argued that such biased retention of duplicated genes were a driving force for morphological complexity, increase in biological diversity and eukaryote adaptive radiation [8, 23].

At the same time SSRs themselves are variations. One striking feature of SSRs is its high mutation rate [24]. It is established that SSRs exhibit a very high expansion/contraction rate, mainly through replication errors caused by DNA polymerase strand slippage [25–27]. A typical insertion/deletion event will add/remove one unit, meanwhile changes of several units have also been observed [28]. Theoretically, shorter units allow for more potential replication slippage events per unit length of DNA [29] and are thus likely to be more unstable and carry higher mutation rates [30–31]. It has also been proved that the bases substitution rate is increased in the SSRs sequences [32–33] as well as in their flanking regions [34]. In view of the above experimental evidences, SSRs can be regarded as mutational hot spots in genome sequences.

The distributions and characteristics of SSRs in plant genomes and their relation with the annotated genome components, mainly as genes sequences (including introns and exons), promoters and transposable elements, have been investigated [35–38]. However, the evolution regularities of SSRs in individual plant genomes and plant kingdom evolution have not been extensively studied. In this paper, we studied the evolution regularities of SSRs in individual plant genome and plant kingdom and expected to shed insights onto the evolution of plant genome sequences.

## Materials and Methods

### 1. Genome sequences

In this study, four dicotyledon species (*Arabidopsis thaliana* Col-0 (*A*. *thaliana*), *Glycine max* (*G*. *max*), *Vitis vinifera* (*V*. *vinifera*), *Solanum lycopersicum* (*S*. *lycopersicum*)), four monocotyledon species (*Brachypodium distachyon* (*B*. *distachyon*), *Oryza sativa* Japonica Group (*O*. *sativa*), *Sorghum bicolor* (*S*. *bicolor*), *Zea mays* (*Z*. *mays*)), one ferm species (*Selaginella moellendorffii* (*S*. *moellendorffii*)), one moss species (*Physcomitrella patens* (*P*. *patens*)), and two algae species (*Chlamydomonas reinhardtii* (*C*. *reinhardtii*), *Volvox carteri* (*V*. *carteri*)) were selected for analysis. To analyze whether the SSR distribution pattern is occurring randomly, the other two ecotypes of *A*. *thaliana* and *Drosophila melanogaster* (*D*. *melanogaster*) were selected for calculation. The genome sequences of *A*. *thaliana* (Col-0), *B*. *distachyon*, *G*. *max*, *O*. *sativa* (*Japonica Group*), *S*. *lycopersicum*, *V*. *vinifera* and *D*. *melanogaster* were downloaded from the National Center for Biotechnology Information (NCBI) genome database (ftp://ftp.ncbi.nlm.nih.gov/genomes/). The genome sequences of *C*. *reinhardtii*, *P*. *patens*, *S*. *moellendorffii*, *S*. *bicolor* and *Z*. *mays* were downloaded from the Ensembl plant database (http://plants.ensembl.org/). The genome sequence of *V*. *carteri* was downloaded from the PlantGDB dababase (http://www.plantgdb.org/). The genome sequences of *A*. *thaliana* ecotypes Ler-0 and Ws-0 were downloaded from http://mus.well.ox.ac.uk/19genomes/fasta/MASKED/. Details showed in S1 Table.

### 2. SSRs analysis

SSRs in these twelve plant genome sequences were harvested with a Perl program specifically developed for this paper (see S1 File: perl_program_for_SSR_analyze.rar). We defined a mononucleotide repeat unit with no less than six ((N)x, x≥6; N: A, T, G or C) and di- to hexnucleotide repeats units with no less than three ((N(2–6))x, x≥3, N: A, T, G or C).

In the percentage analysis of SSRs section, we classified nucleotide combinations according to the principle of complementary base and sequence of nucleotide combination and analyzed the data according to different nucleotide combination groups. In mononucleotide SSRs, we classified adenine (A) repeat SSRs and thymine (T) repeat SSRs as a group; cytosine (C) repeat SSRs and guanine (G) repeat SSRs as another group. In dinucleotide SSRs, twelve nucleotide combinations were classified into four groups, named AT/TA, CG/GC, AC/GT/CA/TG and AG/CT/GA/TC. In trinucleotide SSRs, sixty nucleotide combinations were classified into ten groups, named AAT/ATT/ATA/TAT/TAA/TTA, AAC/GTT/ACA/TGT/CAA/TTG, AAG/CTT/AGA/TCT/GAA/TTC, ATC/GAT/TCA/TGA/ATG/CAT, ACT/AGT/CTA/TAG/GTA/TAC, ACC/GGT/CCA/TGG/CAC/GTG, AGG/CCT/GGA/TCC/CTC/GAG, ACG/CGT/CGA/TCG/GAC/GTC, AGC/GCT/GCA/TGC/CAG/CTG and CCG/CGG/CGC/GCG/GCC/GGC. In this section we chose SSRs groups with the same nucleotide number SSRs units as a whole (100%) in a species.

In the percentage analysis of SSRs based on repeat number section, we chose SSRs containing the same repeat number and having more than 1000 total SSRs number to analyze. We chose SSRs groups with the same repeat number and the same nucleotide number SSRs units as a whole (100%) in a species.

### 3. Cluster analysis

The symmetrized Kullback–Leibler divergence analysis [39], a quantity that measures the difference between two subpopulations p and q was defined as $(\sum_{x}p(x)\text{log}\frac{p(x)}{q(x)}+\sum_{x}q(x)\text{log}\frac{q(x)}{p(x)})\times \frac{1}{2}$, was according to percentage of dinucleotide combination and trinucleotide combination, p(x) and q(x) represent the percentage of the same nucleotide compositions in two species respectively, x represents different nucleotide combinations. All pairs of comparisons between the thirteen genomes were performed (including control). The cluster analysis was performed by using the UPGMA method of MEGA4 software package according to the symmetrised Kullback–Leibler divergence analysis.

## Results

### 1. Genome size and GC content

**Fig 1:**
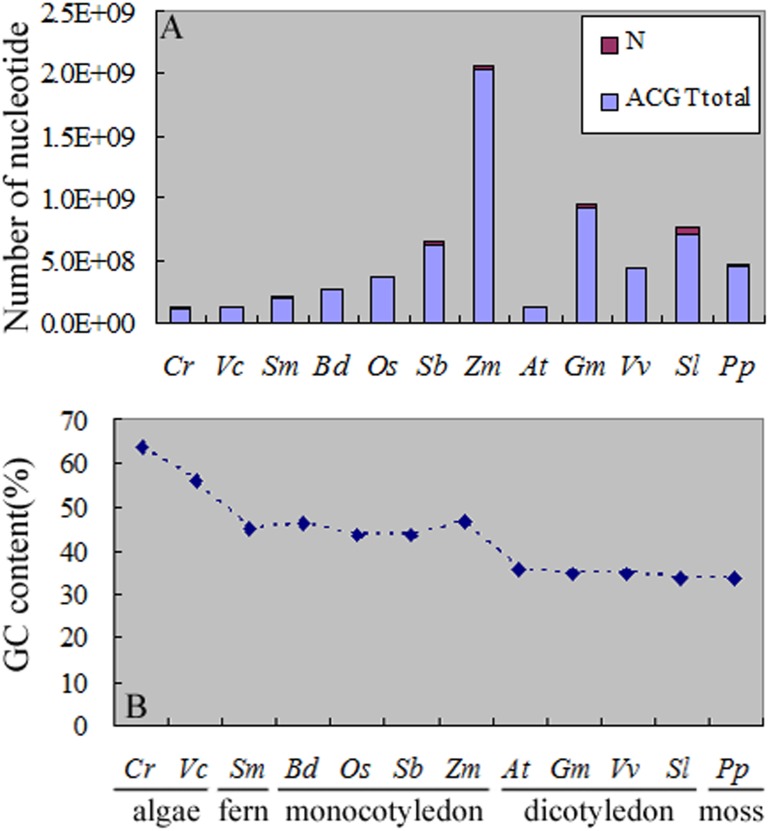
Genome size and GC content in the twelve species studied. (A) Total nucleotide number in the twelve plants genome sequences. (B) The percentage of C/G in the twelve plants genome sequences.

Among these twelve plants the genome sizes of *C*. *reinhardtii* (105,409,962 nucleotides), *V*. *carteri* (125,353,261 nucleotides) and *A*. *thaliana* (118,960,141 nucleotides) (referring to the ecotype Col-0 hereinafter if not labeled) were smaller than the others, and the *Z*. *mays* genome (2,046,695,782 nucleotides) was the largest (Fig 1A). We calculated the nucleotide percentage of these genomes. The percentage of adenine (A) was approximately equal to that of thymine (T) in the twelve plant single-stranded genome sequences. The cytosine (C) and guanine (G) showed the same trend (S1 Fig). In the twelve plants *C*. *reinhardtii* (63.87%) and *V*. *carteri* (56.11%) genomes were GC-rich, and the other genomes were AT-rich (Fig 1B). The GC content in fern *S*. *moellendorffii* (45.23%) and monocotyledon (43.55%~46.89%) were approximately equal and the GC content in moss *P*. *patens* (33.60%) was close to that of dicotyledon (33.95%~36.03%) (Fig 1B).

### 2. Overall SSRs density

**Fig 2:**
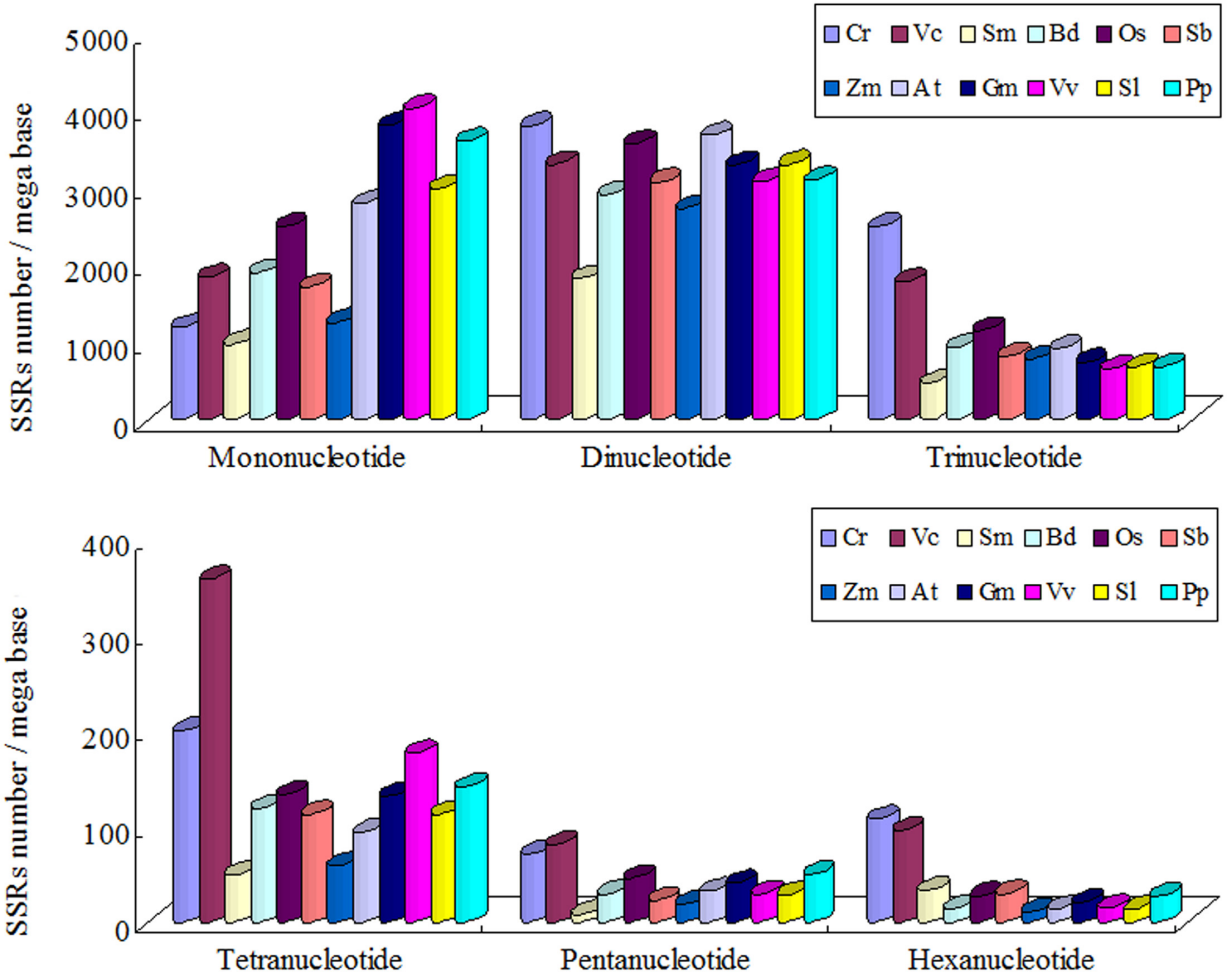
The SSRs density in the twelve plants.

We analyzed the SSRs number and SSRs density (SSRs number / mega bases) in the plant genome sequences (Fig 2 and S2 Table). The densities of mono-, di- and tri- SSRs were significantly higher than other SSRs, so we chose these SSRs as the main SSRs. The densities of mononucleotide SSRs in moss *P*. *patens* and dicotyledon were significantly higher than other plants. The SSRs densities from trinucleotide to hexanucleotide in *C*. *reinhardtii* and *V*. *carteri* were higher than those of other plants, which was consistent with the results of zhao et al. [35]. While the SSRs densities from mononucleotide to pentanucleotide in *S*. *moellendorffii* were lower than other plants.

### 3. The main SSRs analysis among plant genomes

**Fig 3:**
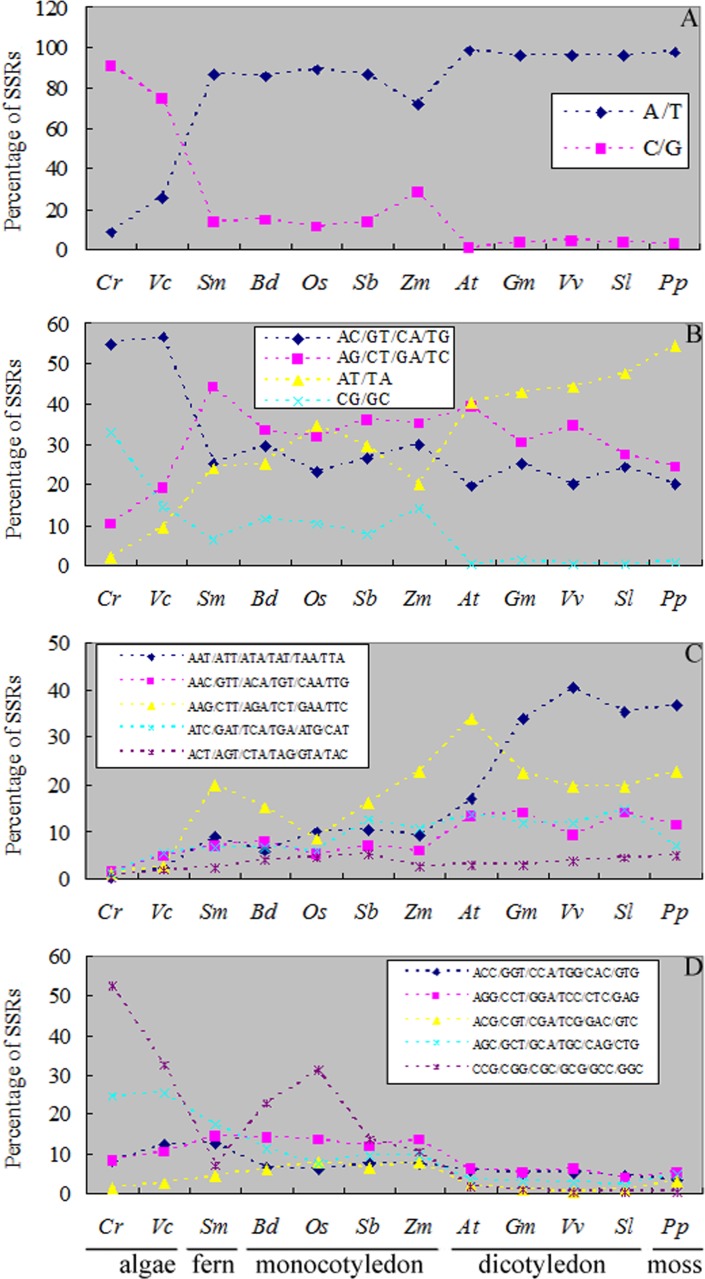
The percentages of SSRs with different combinations in the twelve plant genomes. (A)The SSRs percentage of mononucleotide repeats. (B) The SSRs percentage of dinucleotide repeats. (C) The SSRs percentage of trinucleotide repeats.

We have shown that the mononucleotide, dinucleotide, and trinucleotide repeats were more abundant than the longer repeated units SSRs, so we focused on these three types of SSRs. In mononucleotide SSRs, the A/T percentage was similar between fern *S*. *moellendorffii* (86.15%) and monocotyledon (71.96%~89.17%). While the percentages of A/T in moss *P*. *patens* (97.30%) and dicotyledon (96.01%~98.76%) were approximately equal. There was a special case that *Z*. *mays* had significantly lower A/T (71.96%) than other monocotyledon (85.85%~89.17%). The algae *C*. *reinhardtii* and *V*. *carteri* had the higher C/G content (74.55%~91.07%) (Fig 3A), which was different from other plants in mononucleotide.

In dinucleotide SSRs, the AT/TA percentage increased along with the evolution of plants from algae, fern and monocotyledon to dicotyledon. The CG/GC percentage showed opposite trend. The moss *P*. *patens* was a special case which showed the same trend as dicotyledon (Fig 3B).

In trinucleotide SSRs, the percentages of combination including two or three A/T were in a rising trend along with the evolution of plants from algae, fern and monocotyledon to dicotyledon (Fig 3C). The percentage of CCG/CGG/CGC/GCG/GCC/GGC and AGC/GCT/GCA/TGC/CAG/CTG was more than 57.72% in algae. So the percentages of other trinucleotide combination including two or three C/G decreased only during the terrestrial plants evolution (Fig 3D). However, there were some exceptions. For example, the moss *P*. *patens* showed the same trend with the dicotyledon (Fig 3C and 3D) and the percentage of CCG/CGG/CGC/GCG/GCC/GGC in *O*. *sativa* was significantly higher than other monocotyledon studied in this paper.

### 4. The main SSRs analysis based on repeat number within plant genomes

**Fig 4:**
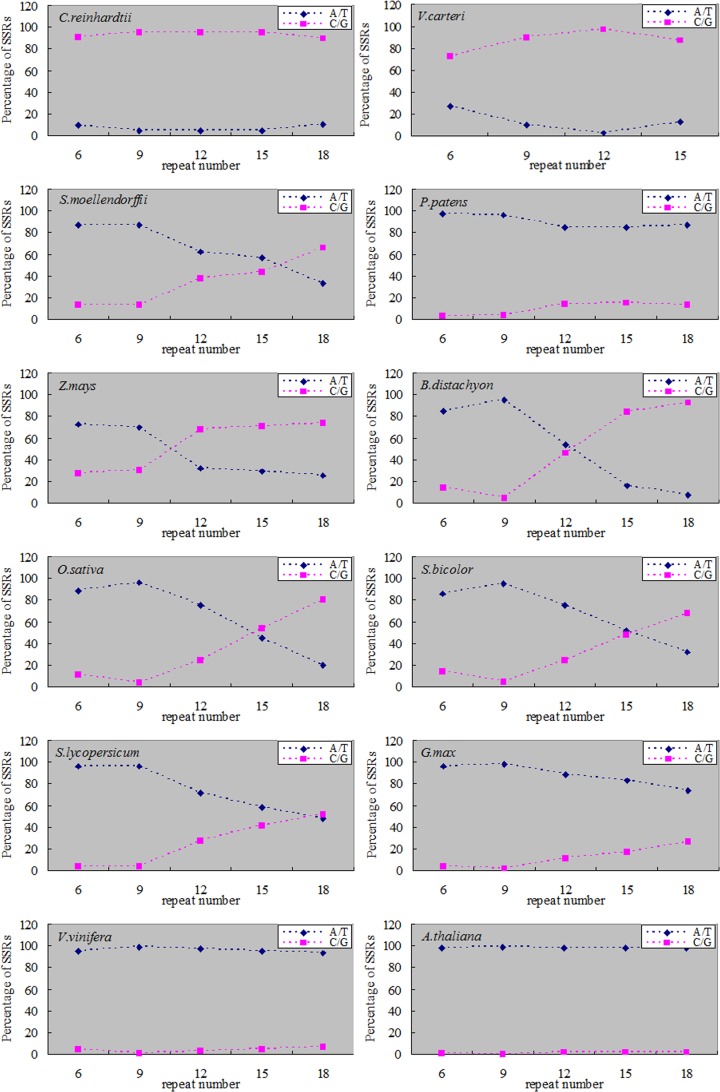
The percentages of mononucleotide SSRs with different repeat number in twelve plant genomes. 6: repeat number 6, 7 and 8; 9: repeat number 9, 10 and 11; etc.

With the increase of the SSRs repeat number, different species showed a different evolutionary trend. In mononucleotide SSRs, the percentage of mononucleotide repeats was different between terrestrial plants and algae. The percentages of mononucleotide repeats had no obvious change with the increase of the repeat number and the percentage of C/G repeats (more than 90%) was obviously higher than that of the A/T repeats in algae *C*. *reinhardtii*. In the monocotyledonous plants and fern, the percentages of A/T repeats decreased along with the increase of the repeat number, and gradually lower than the percentage of C/G repeats at high repeat number. In the dicotyledonous plants and moss, A/T repeats decreased with the increase of repeat number, but the percentages of A/T repeats were always higher than the percentages of C/Gs (Fig 4).

**Fig 5:**
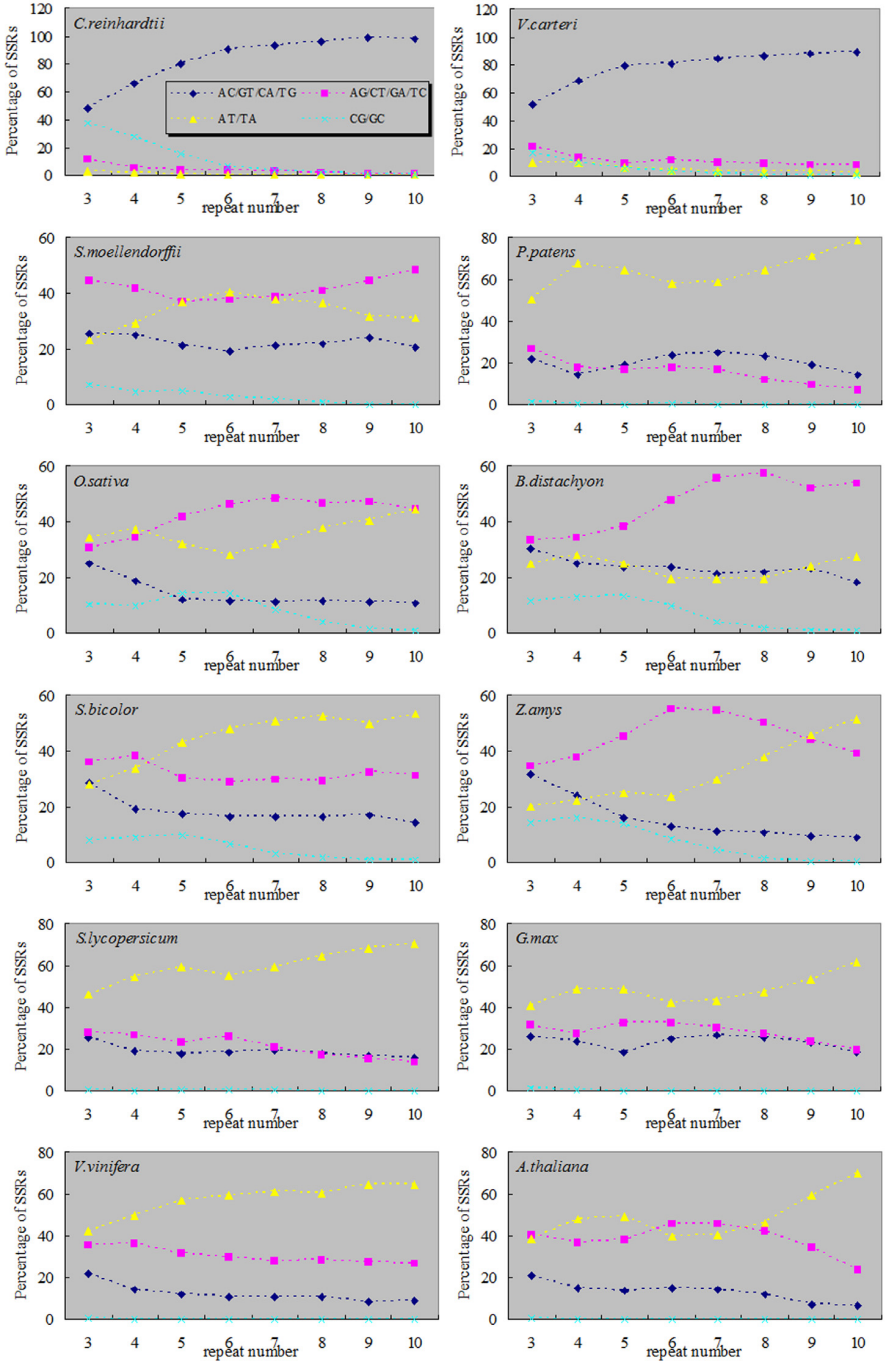
The percentages of dinucleotide SSRs with different repeat number in twelve plant genomes.

In dinucleotide SSRs, algae and terrestrial plants exhibited different patterns as well. In algae, the percentage of AC/GT/CA/TG combination was higher than other dinucleotide combinations, and it showed a significant increase along with the increase of repeat number. On the contrary, in terrestrial plants, the percentage of AC/GT/CA/TG combination decreased along with the increase of repeat number. In terrestrial plants, the percentages of AT/TA combination showed a rising trend along with the increase of repeat number (except for *B*. *distachyon*). Meanwhile, AT/TA combination was dominant in dicotyledon and moss *P*. *patens*. In monocotyledon (except for *S*. *bicolor*) and fern *S*. *moellendorffii*, AG/CT/GA/TC combination was dominant and the percentage increased along with the increase of repeat number. However the percentage of AG/CT/GA/TC combination declined along with the increase of repeat number in dicotyledon and moss *P*. *patens*. The percentages of CG/GC combination decreased along with the increase of repeat number in the twelve plants. Dicotyledon and moss were significantly lower in percentage of CG/GC combination than other plants (Fig 5).

**Fig 6:**
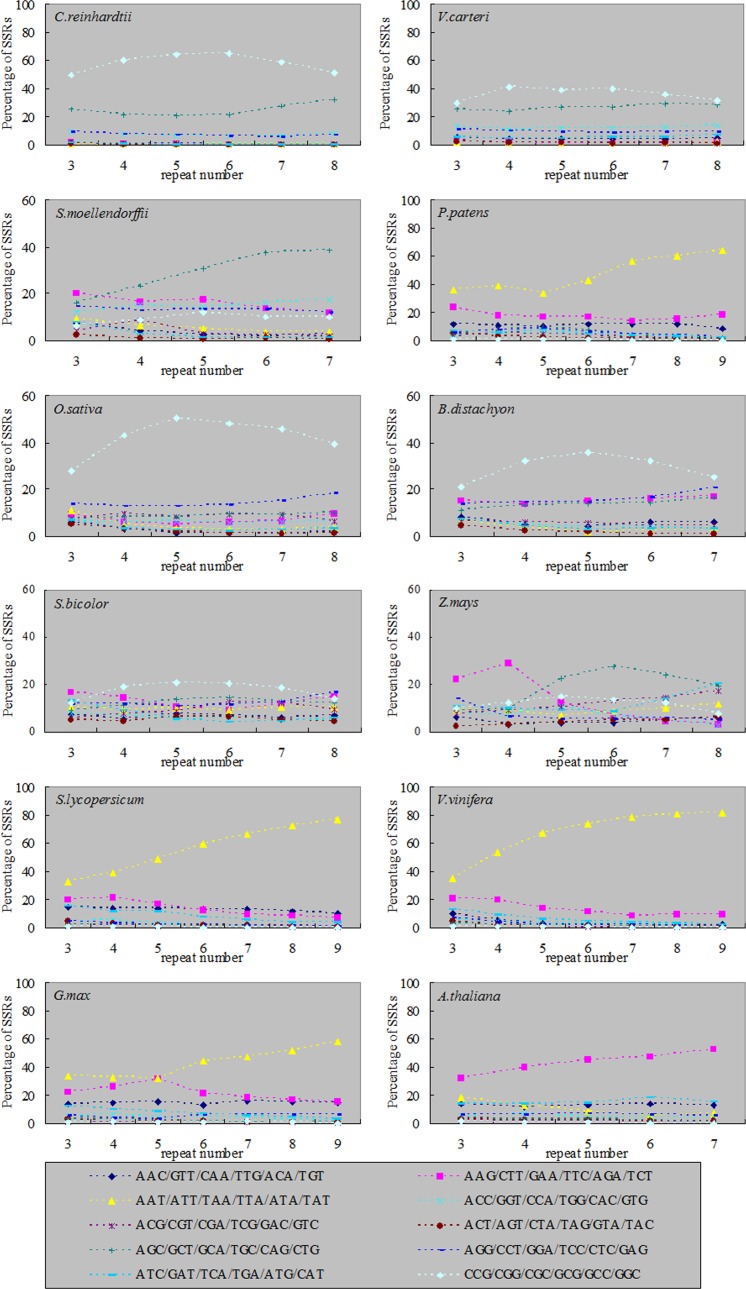
The percentages of trinucleotide SSRs with different repeat number in twelve plant genomes.

In trinucleotide SSRs, the percentages of three nucleotide combinations showed a diversification trend along with the increase of repeat number in the twelve plants. In algae and monocotyledon plants (except for *Z*. *mays*), the combinations of CCG/CGG/CGC/GCG/GCC/GGC were dominant SSRs. In moss *P*. *patens* and dicotyledon (except for *A*. *thaliana*), AAT/ATT/ATA/TAT/TAA/TTA combinations were dominant SSRs, and the percentages increased along with the increase of repeat number. The percentage of SSRs with trinucleotide combinations in *A*. *thaliana* and *Z*. *mays* differed from other plants along with the increase of repeat number (Fig 6).

### 5. Clustering analysis based on SSRs percentage

**Fig 7:**
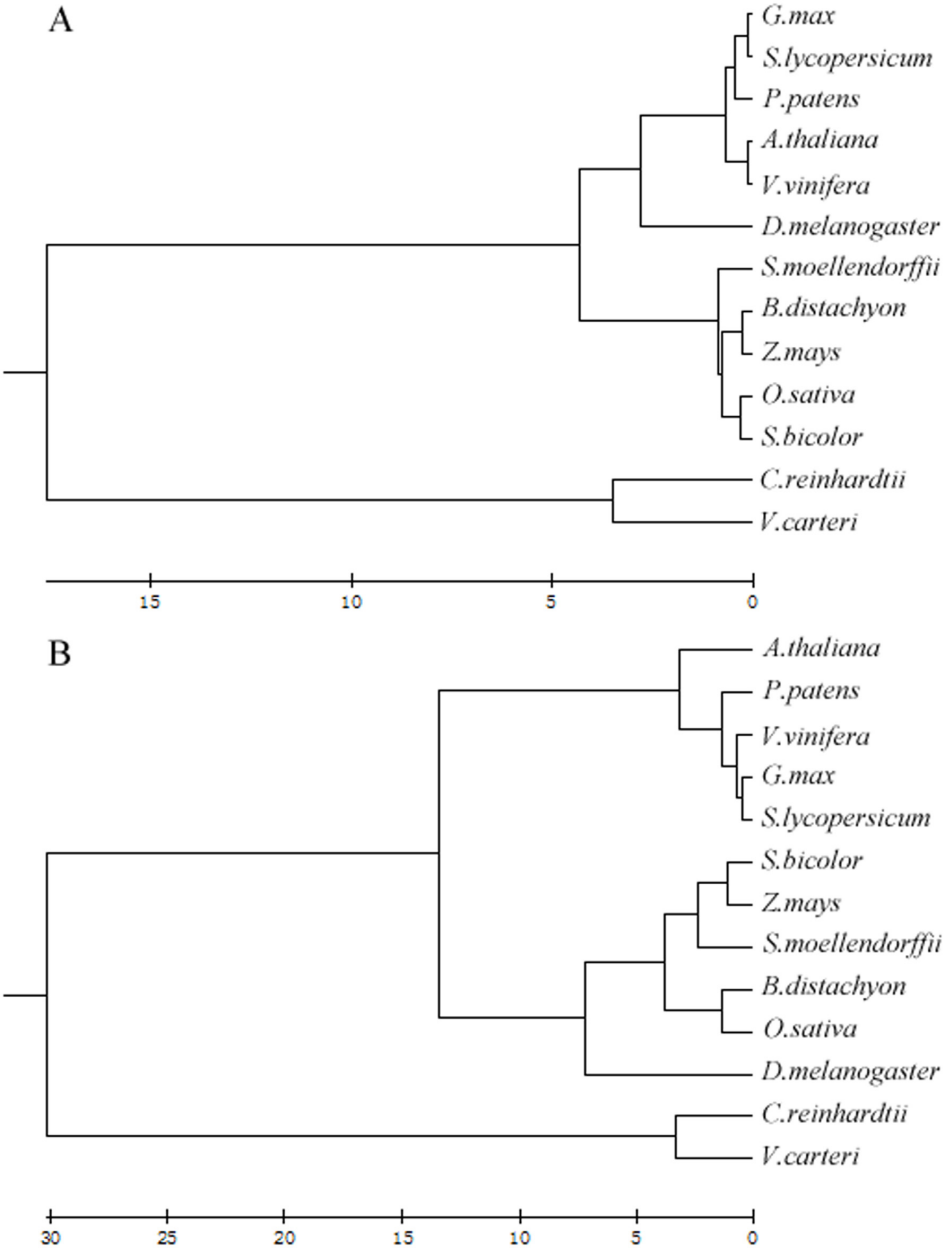
Cluster analysis of SSR percentage based on dinucleotide combination and trinucleotide combination in algal and terrestrial plants genomes. (A) Cluster analysis of dinucleotide combination percentage. (B) Cluster analysis of trinucleotide combination percentage. We chose *D*. *melanogaster* as a control.

SSRs percentage clearly distinguished the algae from the terrestrial plants (Fig 7). Within the twelve plants, a symmetrised Kullback–Leibler divergence analysis based on dinucleotide combinations percentage or trinucleotide combinations percentage also divided the monocotyledonous/fern and dicotyledonous/moss species into two recognizable clades (Fig 7). The relationship between the terrestrial plants was somewhat different when a clustering analysis was applied as an alternative to the symmetrised Kullback–Leibler divergence analysis. Based on dinucleotide combination percentage, fern can separate from monocotyledonous (Fig 7). We chose *D*. *melanogaster* as a control, and found that the GC content in *D*. *melanogaster* genome was comparable to monocotyledon (S2 Table).

## Discussion

### 1. SSRs evolution accompanied by evolution of plant genomes

Plants have undergone the process of evolution which was from aquatic to terrestrial habitats in the living environment, and from simple to complex in morphological structures. In the genome level, plants have gone through huge changes, including the duplications of chromosome fragments and/or whole genomes, loss of chromosome fragments, and so on [19, 40–41]. In this study, we found simple sequence repeats in plant genome sequences have evolutionary regularities relative to the plant genome evolution.

**Fig 8:**
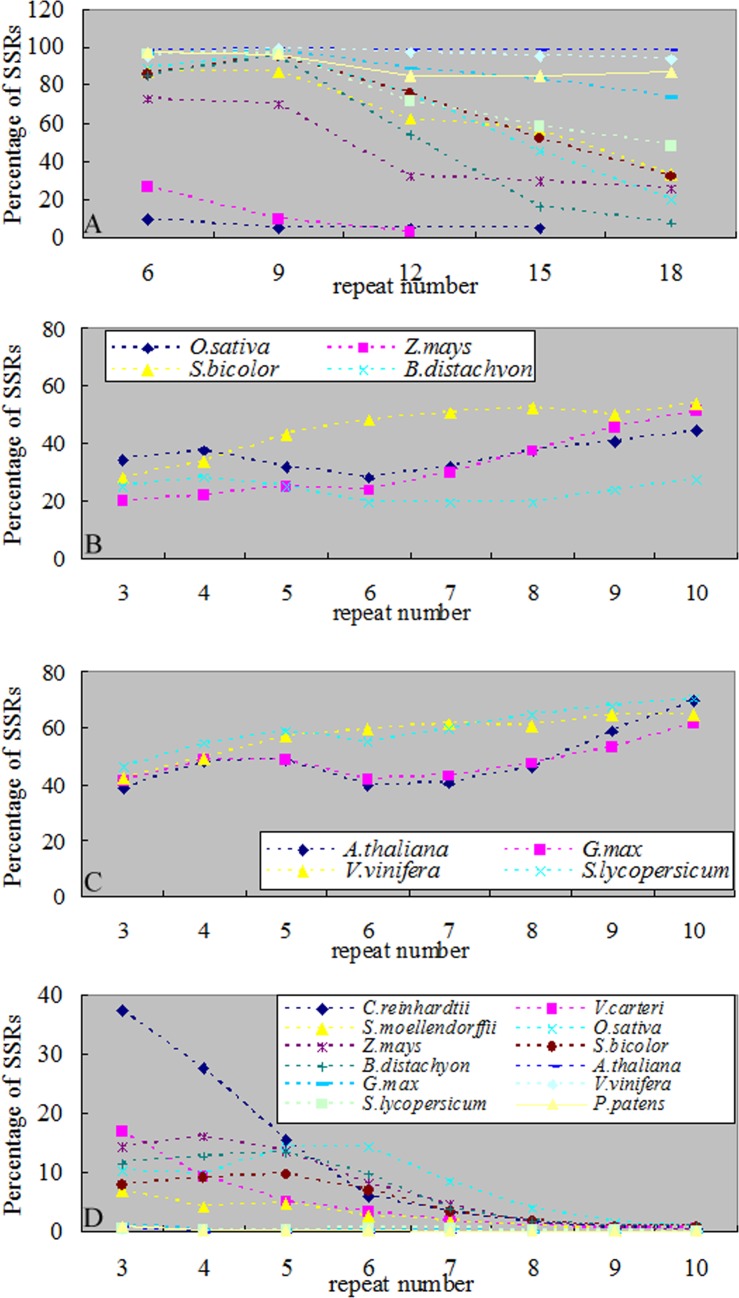
Variation of different combinations of SSRs. (A)The A/T frequency changed with different repeat number in twelve plants genome sequences. (B)The AT/TA frequency changed with different repeat number in monocotyledon genome sequences. (C) AT/TA frequency changed with different repeat number in dicotyledon genome sequences. (D) CG/GC frequency changed with different repeat number in twelve plants genome sequences. Fig8A and D with the same legend.

First, the main SSRs were those that contain combination of repeat units consisting of 1–3 nucleotides in both algae and terrestrial plants (Fig 2). Second, in mononucleotide SSRs, the A/T percentage gradually increased along with the evolution of plants (except for *P*. *patens*) (Fig 3A). This result was consistent with of previous studies [42–44]. With the increase of SSRs repeat number, the percentage of A/T in *C*. *reinhardtii* had no significant changes, while the percentages of A/T in terrestrial plants were gradually declining and the declining trends in monocotyledon were significantly greater than dicotyledon (Figs 4 and 8A). Toth et al. [43] suggested that the poly(A) tails of densely scattered retroposed sequences and processed pseudogenes are responsible for this higher proportion of A/T-rich repeats, which may the evolutionary driver of A/T mononucleotide SSRs. Third, in dinucleotide SSRs, the percentage of AT/TA increased along with the evolution of plants (Fig 3B). In the terrestrial plant, its percentage also increased along with the increase of repeat number (Fig 5), the trends in dicotyledon were even clearer than in monocotyledon (Fig 8B and 8C). The percentage of CG/GC showed the opposite pattern to the AT/TA (Fig 8D). However AC/GT/CA/TG was the most frequent dinucleotide repeat units in all vertebrates and arthropods [43], which was different from the terrestrial plant (S2 Table). Forth, in trinucleotide SSRs, the percentages of combinations including two or three A/T were in a rising trend along with the evolution of plants from algae, fern and monocotyledon to dicotyledon (Fig 3C). Meanwhile, the dominant SSRs were differentiated in different species with the increase of repeat number. For example, algae and monocotyledon (except for *Z*. *mays*) preferentially chose CCG/CGG/CGC/GCG/GCC/GGC as dominant SSRs, moss *P*. *patens* and dicotyledon (except for *A*. *thaliana*) chose AAT/ATT/ATA/TAT/TAA/TTA as dominant SSRs (Fig 6). It is worth noting that ACG/CGT/CGA/TCG/GAC/GTC and ACT/AGT/CTA/TAG/GTA/TAC were low frequency in most plants and animals [43–44]. Our results clearly demonstrate that the dominant SSR types are taxon-dependent.

Toth et al. [43] thought that strand-slippage theories alone cannot explain microsatellite distribution in the genome as a whole, enzymes and other proteins involved in various aspects of DNA-processing (i.e., replication and repair) and chromatin remodeling may be responsible for the taxon-specificity of microsatellite abundance. Harr et al. [45] thought that the mismatch repair system may have an important role in shaping genome composition.

### 2. Algae showed different regularities of SSRs from terrestrial plants

*C*. *reinhardtii* is a unicellular green algae whose lineage diverged from terrestrial plants over one billion years ago. Many *C*. *reinhardtii* and angiosperm genes are derived from ancestral green plant genes [46]. Genes shared by *C*. *reinhardtii* and animals are derived from the last plant-animal common ancestor and many of these have been lost in angiosperms [47]. *C*. *reinhardtii* also displays extensive metabolic flexibility under the control of regulatory genes that allow it to inhabit distinct environmental niches and to survive fluctuations in nutrient availability [48]. This may account for that fact that the GC content (Fig 1B) and SSRs characteristics (Figs 3–6) were different between *C*. *reinhardtii* and terrestrial plant genome sequences.

### 3. *Physcomitrella patens* SSRs exhibit a specific distribution pattern

The haploid moss *P*. *patens* is a paleopolyploid. The genome sequences and construction of linearized phylogenetic trees suggest that a large-scale duplication, possibly involving the whole genome, has occurred between 30 and 60 million years ago [49]. Gene ontology and pathway association of the duplicated genes in *P*. *patens* revealed different biases of gene retention compared with seed plants [19, 49]. We found the characteristics of SSRs in *P*. *patens* genome sequences were obviously different from *C*. *reinhardtii* (Figs 1–6). *P*. *patens* is the earliest terrestrial plant. During the adaptation of the terrestrial environment, great changes have occurred in the structure and function, for example desiccation tolerance, auxin, ABA, cytokinin signaling, and so on [19]. These changes are based on the changes in the genome sequences [19, 49]. SSRs differences between *P*. *patens* and *C*. *reinhardtii* may reflect the changes to some extent.

Surprisingly, we discovered that *P*. *patens* shared the same characteristics of SSRs with dicotyledon (Figs 1–6). However, in comparison with the dicotyledon, *P*. *patens* possessed more tetranucleotide (except *V*. *vinifera*), pentanucleotide and hexanucleotide SSRs (Fig 2). DNA polymerase strand slippage was a major factor of SSRs chain extension [25–27]. The different characteristics of SSRs may reflect the different fidelity of DNA polymerase between *P*. *patens* and dicotyledon. Of course, further experiments are required to prove this hypothesis.

### 4. Monocotyledon and dicotyledon SSRs analysis

All flowering plants have survived at least three large-scale duplications/diploidizations over the last 300 million years [23]. The monocotyledon branched off from dicotyledon 140~150 million years ago [50]. In the monocotyledon and dicotyledon genome sequences the percentage of A/T are higher than C/G’s and the dicotyledon has higher A/T percentage than monocotyledon (Fig 1B). But there are special cases that the regularities of SSR variation are different from other closely related plants due to their specific changes in the genome sequences.

Our results showed that the percentages of SSRs in *Z*. *mays* genome sequences, from mononucleotide to hexanucleotide combination (except for trinucleotide) were lower than other monocotyledon plants in this paper (Fig 2). In detail, the frequencies of mononucleotide and dinucleotide combinations, which consist of A/T, were lower than other monocotyledon plants studied in this paper (Fig 3A and 3B). The *Z*. *mays* genome has undergone several rounds of genome duplication [14, 41]. Then the size of *Z*. *mays* genome has expanded dramatically (to 2.3 gigabases) (Fig 1A) over the last ~3 million years via a proliferation of long terminal repeat retrotransposons [51], which rarely contain SSRs [52] and show a tendency to insert into some SSRs, such as AT-rich repeats [53–54]. These genome changes can thus lead to a significant decrease in the percentage of SSRs.

The percentage of AG/CT/GA/TC and AAG/CTT/AGA/TCT/GAA/TTC combinations in *A*. *thaliana* were higher than other studied dicotyledons (Figs 3B, 3C and 6). The *A*. *thaliana* genome has undergone large-scale gene duplications or even duplications of the entire genome followed by subsequent the high percentage of gene loss and extensive local gene duplications (Fig 1A) [11, 40]. These combinations maybe retained in the process of the evolution.

### 5. SSRs comparative analysis between different ecotype plants

As we all know that SSRs are highly polymorphic. SSRs are already widely used in genetic diversity analysis and evolutionary analysis of species, and have been widely used in crop molecular assisted breeding [55–59]. In this paper we mainly analyzed the SSR difference in/among species. At the same time we analyzed the genome sequences of three *A*. *thaliana* common ecotypes (*Columbia* (Col-0), *Landsberg erecta* (Ler-0) and *Wassilewskija* (Ws-0)). We found there were different SSRs regularities among three ecotypes. But the differences within the three ecotypes are smaller than that between species (S2 Table).

## Conclusion

With the evolution of plants and plant genomes, SSRs located in chromosome also undergone regular changes. The percentages of SSRs, which (mainly) consist of C/G, were gradually declining. And the percentages of SSRs, which (mainly) consist of A/T, were gradually increased. At the same time, for a particular species, SSRs composition and percentage were changed accompanied by the genome/genes varies (duplication, polyploidy and deletion). Thus the regularities of SSRs in the twelve plant genome sequences can provide clues for revealing the evolution of plant genomes.

Given the current of sequenced plant genome restrictions, fern and moss chose only one species, in the paper we cannot large sample analysis of SSRs feature in different evolutionary position plants.

## Supporting Information

S1 Fig The percentage of four nucleotides in the twelve plants genome. (TIF)

S1 File perl_program_for_SSR_analyze. (RAR)

S1 Table The plant genome seqence files download link. (XLS)

S2 Table The twelve plant genome and Arabidopsis thaliana different ecotype raw data. (XLS)
