# Supplementary figures and images for: Evolution Analysis of Simple Sequence Repeats in Plant Genome
Source: PLoS One. 2015 Dec 2;10(12):e0144108. doi: 10.1371/journal.pone.0144108 (PMC4668000; doi:10.1371/journal.pone.0144108)

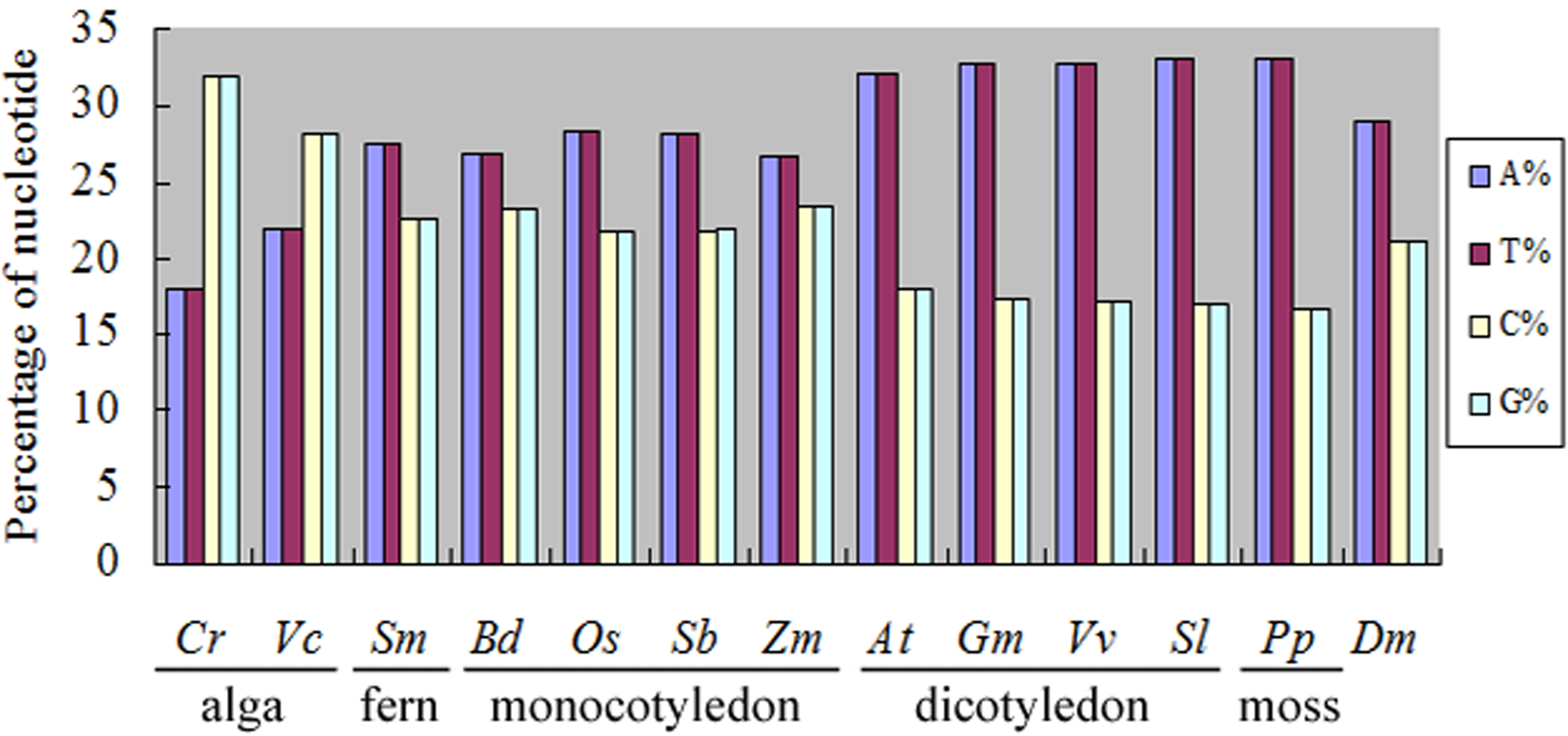

Supplement: S1 Fig — (TIF) [file pone.0144108.s001.tif]
